# Lattice innovative flow diverter with mechanical balloons: a new breakthrough and efficacy evaluation in the treatment of unruptured intracranial aneurysms

**DOI:** 10.3389/fmed.2025.1612754

**Published:** 2025-09-10

**Authors:** Xufeng Sun, Yueyang Peng, Bin Li, Mengyuan Zhang, Zhifeng Wen

**Affiliations:** ^1^Department of Neurosurgery, The First Hospital of China Medical University, Shenyang, China; ^2^Fuxin People’s Hospital (Fuxin Women and Children’s Medical Center), Fuxin, China; ^3^Department of Information Center, The First Hospital of China Medical University, Shenyang, China; ^4^Department of Neurology, The First Hospital of China Medical University, Shenyang, China

**Keywords:** Lattice FD-MB, unruptured intracranial aneurysms, retrospective study, therapeutic effect, safety profile

## Abstract

**Purpose:**

The Lattice Innovative Flow Diverter with Mechanical Balloons (Lattice FD-MB) provides superior positioning accuracy and wall apposition during deployment, potentially addressing the limitations of traditional mesh stents in treating complex lesions. However, clinical data on the efficacy and safety of the Lattice FD-MB for unruptured intracranial aneurysms (UIAs) remain limited. This study evaluates the effectiveness and safety of the Lattice FD-MB in UIA treatment.

**Methods:**

We retrospectively analyzed 29 UIA patients treated with the Lattice FD-MB at our institution between October 2023 and October 2024. After the operation, digital subtraction angiography (DSA) and computed tomography angiography (CTA) were performed to assess aneurysm occlusion. Clinical follow-up, including modified Rankin scale (mRS) scores and DSA, was conducted at 3, 6, and 12 months postoperatively.

**Results:**

The Lattice FD-MB was successfully deployed in all patients, achieving a 100% procedural success rate with no intraoperative device-related failures. Intraoperative complications occurred in two cases (6.9%). Postoperatively, 27 patients (93.1%) showed favorable clinical outcomes, defined as no new neurological deficits or hemorrhagic/ischemic events. At discharge, functional outcomes (mRS) were as follows: mRS 0 (no symptoms) in 23 patients (79.3%), mRS 1 (no significant disability) in 3 patients (10.3%), and mRS 2 (slight disability) in 3 patients (10.3%). On follow-up, the proportion of patients with mRS 0 increased over time, with 82.8% (24/29) at 3 months, 86.2% (25/29) at 6 months, and 89.7% (26/29) at 12 months, demonstrating sustained clinical stability and neurological improvement.

**Conclusion:**

The Lattice FD-MB appears to be a feasible and safe treatment for UIAs, with promising patient outcomes. Further research is warranted to investigate prognostic factors influencing long-term results after Lattice FD-MB treatment.

## Introduction

Unruptured intracranial aneurysms (UIAs) are a common cerebrovascular condition, with imaging studies estimating their prevalence in the general population at 2.8–7.0% (1–3). Recent advancements in non-invasive imaging, particularly high-resolution magnetic resonance angiography (HR-MRA) and 3D rotational angiography, along with the increasing adoption of routine health screenings, have significantly improved the early detection of asymptomatic UIAs. Consequently, the number of diagnosed cases has steadily increased, posing new challenges in clinical management, particularly in balancing intervention risks against the potential for aneurysm rupture (4).

The primary concern with UIAs is their unpredictable rupture risk, which can lead to subarachnoid hemorrhage, a catastrophic event with a 50–60% mortality rate due to acute bleeding or secondary complications such as cerebral vasospasm and rebleeding. Among survivors, over 30% experience permanent disabilities, including motor deficits, cognitive impairments, and long-term psychosocial consequences (5, 6). These outcomes highlight the critical need for timely diagnosis and risk-stratified treatment strategies. UIAs could also present with non-specific symptoms, such as subtle cognitive decline, mood disturbances, or transient neurological deficits, that are easily overlooked (7). While not pathognomonic, these manifestations warrant heightened clinical suspicion, especially in high-risk populations.

Over the past few decades, the management of intracranial aneurysms has undergone significant evolution, driven by advances in treatment paradigms and emerging technologies (8). Currently, surgical clipping and endovascular therapy remain the primary treatment options (9). Surgical clipping, a well-established neurosurgical procedure, involves the direct occlusion of the aneurysm neck through a craniotomy to eliminate blood flow into the aneurysm sac. While highly effective, its invasiveness and associated risks may limit its applicability in certain patients. In contrast, endovascular therapy, particularly coiling, has gained widespread acceptance due to its minimally invasive nature, shorter hospital stays, and lower perioperative morbidity. Randomized trials and meta-analyses have demonstrated that endovascular coiling provides superior clinical outcomes, including fewer procedure-related complications and better functional recovery, ultimately improving patients’ quality of life (10–13).

Despite these advancements, treatment of complex intracranial aneurysms, such as those with irregular morphology, critical anatomical locations, or wide necks, remains a challenge. Traditional endovascular techniques often fail to achieve durable occlusion in such cases, increasing the risks of recanalization, coil compaction, or parent artery compromise. Against this background, the Lattice Innovative Flow Diverter with Mechanical Balloons (Lattice FD-MB) has been developed (14). This next-generation device integrates a mechanically expandable balloon system with a high-density mesh stent, enabling real-time controlled deployment, optimal wall apposition, and precise vessel alignment—features that are critical for managing anatomically challenging aneurysms. By combining flow diversion with dynamic mechanical support, the Lattice FD-MB is designed to overcome the limitations of conventional flow diverters, such as incomplete expansion or migration in tortuous vasculature. Given the unmet clinical need in treating complex UIAs, this study aims to evaluate its technical success, aneurysm occlusion efficacy, and safety profile in a real-world cohort, providing evidence for its clinical application in challenging aneurysms.

## Methods

### Study design and patient population

This retrospective study included patients with UIAs treated with the Lattice FD-MB at the Department of Neurosurgery, First Affiliated Hospital of China Medical University, between October 2023 and October 2024. The eligibility criteria included the following: (1) age 18–80 years; (2) radiologically confirmed UIA; and (3) saccular, fusiform, or dissecting aneurysm morphology.

The exclusion criteria were as follows: (1) age <18 years or >80 years; (2) life expectancy <3 years due to severe comorbidities (e.g., terminal malignancy, end-stage organ failure); (3) ruptured, mycotic, or infected aneurysms; (4) severe intracranial atherosclerotic stenosis (>70% lumen reduction) adjacent to the aneurysm; (5) known hypersensitivity to iodinated contrast media or cobalt-chromium alloys; and (6) contraindications to antiplatelet/anticoagulant therapy.

The Institutional Review Board of the First Affiliated Hospital of China Medical University approved this study, which was conducted in compliance with the Declaration of Helsinki. All participants provided written informed consent before enrollment.

### Endovascular procedure

The Lattice FD-MB implantation protocol consisted of six key steps as listed below:

1. Device selection: Vessel dimensions and required landing length were measured using preoperative angiography. The Lattice model-selection algorithm was employed to determine appropriate device sizing, with final selection guided by lesion characteristics and operator judgment.
2. Access establishment: Following femoral artery access, the delivery system was introduced through an 8F guiding sheath. The microcatheter and guidewire were navigated to position the undeployed stent at the target landing zone under fluoroscopic guidance.
3. Stent deployment: Controlled deployment was achieved through simultaneous application of forward pressure on the pusher rod and gradual microcatheter withdrawal. Initial stent fixation was then confirmed by the formation of the characteristic proximal ‘goblet’ configuration.
4. Balloon-assisted apposition: Mechanical balloons were sequentially inflated within the stent lumen to optimize wall apposition and to ensure complete scaffold expansion (2–3 balloons remained partially exteriorized during positional adjustments).
5. Retrieval protocol (if needed): The stent was retrieved into the protective sheath when the microcatheter tip was distal to the retrieval markers.
6. Final deployment: The tail end was confirmed to be fully expanded before detachment, with the delivery system retrieved.

### Data collection and outcome assessment

Comprehensive patient data were collected through a systematic review of electronic medical records, preoperative and postoperative imaging archives, and follow-up angiographic assessments. Extracted variables included the following: (1) Demographic characteristics: age, sex, and vital signs, such as body temperature, pulse rate, respiratory rate, and systolic/diastolic blood pressure, at admission; (2) Clinical history: presenting symptoms, prior cerebrovascular events, comorbidities (hypertension and diabetes mellitus), dyslipidemia [triglycerides, total cholesterol, High-Density Lipoprotein Cholesterol (HDL-C), and Low-Density Lipoprotein Cholesterol (LDL-C)], cardiac diseases, allergies, smoking status, alcohol consumption, and family history of aneurysms or cerebrovascular diseases; (3) Aneurysm morphology: aneurysm location, morphological classification, and dimensions (aneurysm neck width, dome diameter, and dome-to-neck ratio); (4) Procedure details: Lattice FD-MB specifications (stent diameter and length), use of adjunctive techniques (coil embolization and balloon remodeling), and intraoperative complications; and (5) Outcomes: (i) aneurysm occlusion status was assessed using the Raymond-Roy Occlusion Classification (RROC), a validated scale for evaluating aneurysm occlusion after endovascular treatment (15). RROC Class 1 indicates complete occlusion (no contrast filling in the aneurysm), Class 2 indicates residual neck (contrast filling only in the neck without sac filling), and Class 3 indicates residual sac (contrast filling in the aneurysm sac). All 29 patients underwent DSA evaluation for imaging follow-up at 3, 6, and 12 months postoperatively; (ii) functional outcomes quantified by mRS scores at discharge and follow-up; (iii) intraoperative and postoperative complications; and (iv) device-related parameters (stent diameter, length, and deployment success). All angiographic images were independently reviewed by two board-certified neuroradiologists with 8 and 12 years of experience in cerebrovascular imaging.

Both radiologists were blinded to patient clinical outcomes and treatment details. In cases of disagreement (≤5% of assessments), a third senior neuroradiologist with 15 years of experience adjudicated to reach a consensus. This blinded, independent review process ensured objectivity in evaluating aneurysm occlusion and device positioning.

### Statistical analysis

Continuous variables were expressed as mean ± standard deviation (SD), and categorical variables as frequencies and percentages. Data analysis was conducted using IBM SPSS Statistics for Windows, Version 26.0 (IBM Corp., Armonk, NY, United States).

## Results

### Patient characteristics

The study population comprised 29 patients (mean age: 57.6 ± 9.2 years; 55.2% female). Baseline demographic and clinical characteristics revealed the following:

1. Risk factors: Current/recent smoking history was 34.5% (*n* = 10), and regular alcohol consumption was 17.2% (*n* = 5).
2. Presenting symptoms: Dizziness was 34.5% (*n* = 10), headache was 27.6% (*n* = 8), incidental/asymptomatic was 31.0% (*n* = 9), and rare neurological symptoms (speech incoordination, limb weakness, and visual impairment) were ≤6.9% each.
3. Medical history and comorbidities: Prior cerebrovascular events, including two patients (6.9%) with hemorrhagic stroke and another two (6.9%) with ischemic stroke. Moreover, common conditions included 18 patients (62.1%) with hypertension and 10 patients (34.5%) with dyslipidemia.
4. Aneurysm characteristics: A total of 10 aneurysms (34.5%) were located in the left internal carotid artery (L-ICA) and 10 (34.5%) in the right ICA (R-ICA). Morphologically, saccular aneurysms were the most prevalent (22 aneurysms, 75.9%), followed by dissecting aneurysms (4 aneurysms, 13.8%). Additional procedural and demographic details are summarized in Table 1.

**Table 1:** Baseline characteristics of included patients.

Variables	*N* (%)
Age (years)	57.62 ± 9.16
Female (*n*, %)	16 (55.2%)
Body temperature (°C)	36.46 ± 0.15
Pulse rate	83.41 ± 11.17
Respiratory rate	18.07 ± 1.07
SBP (mmHg)	126.76 ± 19.70
DBP (mmHg)	76.59 ± 11.21
Smoking history (*n*, %)	10 (34.5%)
Alcohol intake (*n*, %)	5 (17.2%)
Presenting symptoms (*n*, %)	
Headache	8 (27.6%)
Dizziness	10 (34.5%)
Incoherent speech	2 (6.9%)
Weakness in the right lower limb	1 (3.4%)
Decreased vision	1 (3.4%)
None	9 (31.0%)
Prior cerebrovascular events (*n*, %)	
Hemorrhagic stroke	2 (6.9%)
Ischemic stroke	2 (6.9%)
Cardiac diseases (*n*, %)	1 (3.4%)
Hypertension (*n*, %)	18 (62.1%)
Dyslipidemia (*n*, %)	10 (34.5%)
TG (mmol/L)	1.87 ± 2.02
TC (mmol/L)	4.21 ± 0.98
HDL (mmol/L)	1.18 ± 0.34
LDL (mmol/L)	2.41 ± 0.88
Diabetes mellitus (*n*, %)	3 (10.3%)
FBG (mmol/L)	5.33 ± 0.88
History of allergies	6 (20.7%)
Aneurysm location (*n*, %)	
L-ICA	10 (34.5%)
R-ICA	10 (34.5%)
L-VA	2 (6.9%)
R-VA	3 (10.3%)
BA	1 (3.4%)
L-ICA and R-ICA	1 (3.4%)
L-VA and R-ICA	1 (3.4%)
Unclear	1 (3.4%)
Morphological classification (*n*, %)	
C5	4 (13.8%)
C6	7 (24.1%)
C7	9 (31.0%)
V4	5 (17.2%)
Multiple	2 (6.9%)
Unclear	2 (6.9%)
Shape of aneurysm (*n*, %)	
Dissecting	4 (13.8%)
Saccular	22 (75.9%)
Both	1 (3.4%)
Unclear	2 (6.9%)
Aneurysm neck width (mm)	4.11 ± 1.05
Dome diameter (mm)	4.85 ± 2.78

### Treatment strategies, and clinical and angiographic follow-up

A range of treatment strategies was used in the study, with some intraoperative complications observed. Postoperatively, most patients had favorable initial conditions, and their recovery trended positively over time. However, individual outcomes required ongoing follow-up and close monitoring (Table 2).

**Table 2:** Treatment strategies and angiographic and clinical outcomes of UIAs treated with Lattice FD-MB.

Variables	*N* (%)
Coil embolization (*n*, %)	6 (20.7%)
Balloon remodeling (*n*, %)	3 (10.3%)
Intraoperative complications (*n*, %)	2 (6.9%)
Massage (*n*, %)	1 (3.4%)
Clinical outcomes	
Postoperative symptom improvement (*n*, %)	27 (93.1%)
mRS 0 at discharge (*n*, %)	23 (79.3%)
mRS 0 at 3 months (*n*, %)	24 (82.8%)
mRS 0 at 6 months (*n*, %)	25 (86.2%)
mRS 0 at 12 months (*n*, %)	26 (89.7%)

Specific treatment methods included the following: (1) coil embolization in six patients (20.7%), a standard endovascular intervention that reduces blood flow into the aneurysm by filling its cavity with coils, promoting thrombus formation and lowering rupture risk; (2) balloon remodeling in three patients (10.3%), which aids vessel dilation and facilitates stent placement; and (3) balloon massage in one patient (3.4%) during the procedure to improve stent-vessel wall apposition and stabilize hemodynamics.

Intraoperative complications occurred in two patients (6.9%), both related to vascular anatomy requiring adjunctive balloon dilation rather than device failure. In the first case, the target vessel segment had pre-existing moderate stenosis, which was dilated using a 3.0 mm × 15 mm balloon to optimize luminal diameter before stent deployment—a planned adjunctive step to ensure adequate stent apposition. In the second case, post-deployment angiography revealed mild focal narrowing at the distal stent edge, which was resolved with gentle balloon dilation to enhance wall apposition. Both complications were transient, managed intraoperatively without sequelae, and did not affect procedural success or postoperative outcomes.

Postoperatively, 27 patients (93.1%) achieved good recovery, with physiological indicators gradually returning to normal. One patient showed signs of improvement with stable conditions. However, the postoperative status of one patient was not reported, which could limit the comprehensiveness of treatment effectiveness evaluation.

At the time of discharge, functional recovery was assessed using the mRS. A total of 23 patients (79.3%) achieved an mRS score of 0, indicating complete recovery with no neurological deficits. The remaining six patients (three with mRS 1 and three with mRS 2) exhibited distinct imaging and clinical outcomes, which were correlated with their mRS scores as follows: (1) aneurysm shrinkage: two patients (both with mRS 1) showed reduced aneurysm size. One involved an ophthalmic artery aneurysm, and the other was a large posterior communicating artery aneurysm located at a vascular curvature. The shrinkage, associated with favorable hemodynamic modifications, aligned with their mild functional impairment, reflecting a positive treatment response; (2) no change in aneurysm size: two patients (one with mRS 1 and one with mRS 2) had no significant change in aneurysm dimensions. One was an embryonic posterior communicating artery aneurysm, where prolonged contrast retention (compared to immediate post-procedural imaging) indicated altered intra-aneurysmal hemodynamics. The other involved basilar artery elongation and dilation, a complex lesion type that likely contributed to the higher mRS score in this case due to persistent vascular instability; (3) in-stent stenosis: one patient (mRS 2) developed mild in-stent stenosis. Despite no history of hyperlipidemia or diabetes, this patient had a long-term smoking history, a known risk factor for vascular endothelial dysfunction, which could have contributed to the stenosis and mild functional disability; (4) aneurysm enlargement: one patient (mRS 2) with a V4-segment dissecting aneurysm experienced enlargement due to incomplete stent coverage, leading to sustained abnormal hemodynamics. This anatomical insufficiency directly correlated with their mild disability. During follow-up at 3, 6, and 12 months post-surgery, the proportion of patients with an mRS score of 0 increased progressively (82.8, 86.2, and 89.7%, respectively). This upward trend reflects continuous neurological recovery over time, confirming the gradual and sustained therapeutic benefits of the Lattice FD-MB intervention.

**Figure 1:**
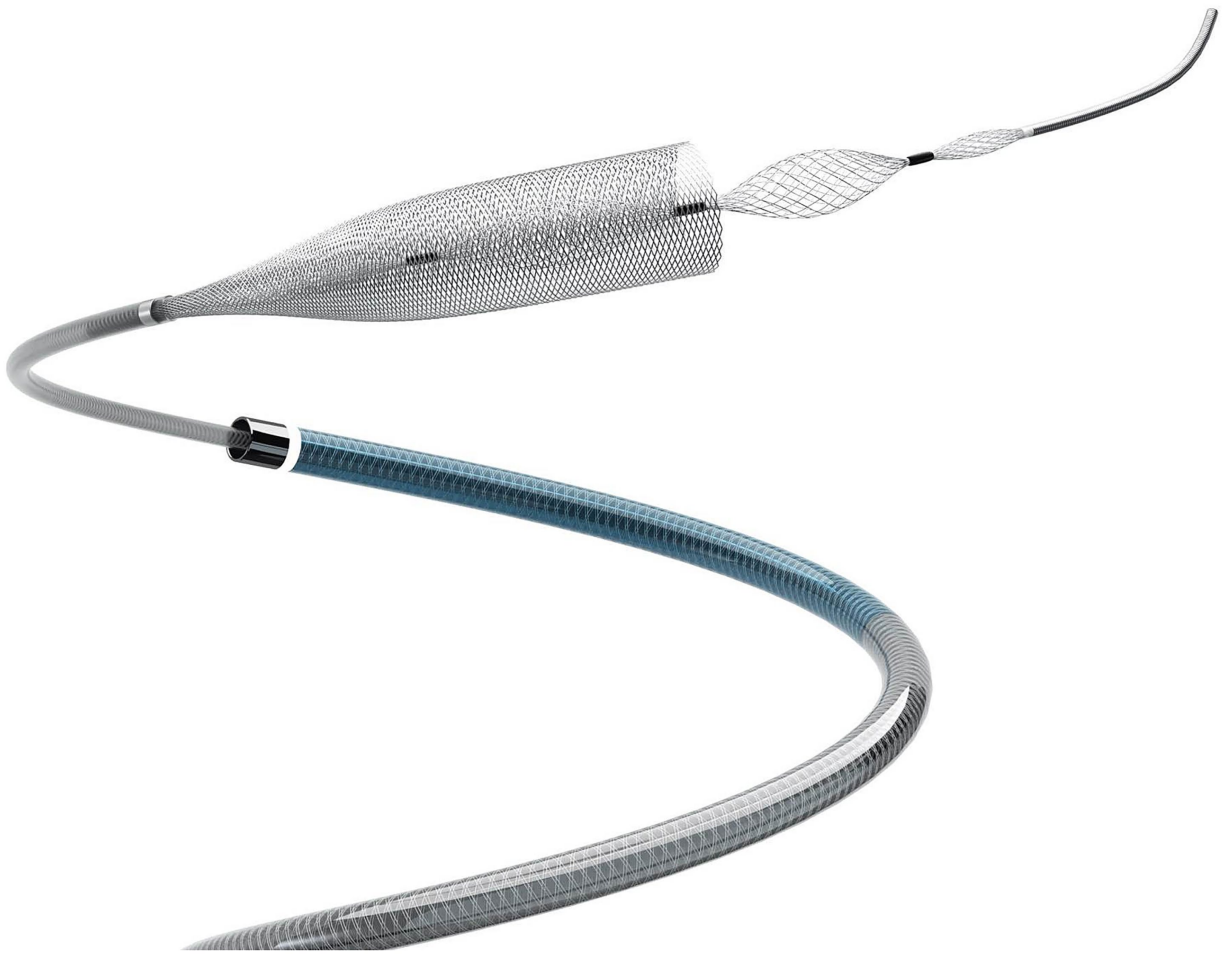
Product diagram of Lattice innovative flow diverter with mechanical balloons (Lattice FD-MB).

At 6 ± 3 months postoperatively, the angiographic outcomes by RROC were as follows: Class 1 (complete occlusion) in 21 patients (72.4%); Class 2 (residual neck) in 5 patients (17.2%); and Class 3 (residual sac) in 3 patients (10.3%). At the 12-month follow-up, Class 1 occlusion increased to 25 patients (86.2%), with 3 patients (10.3%) remaining in Class 2 and 1 patient (3.4%) remaining in Class 3, reflecting progressive occlusion over time. This progressive occlusion pattern is consistent with the delayed thrombotic effects of flow diversion, where neointimal coverage and hemodynamic remodeling typically require 6–12 months to achieve optimal results (see Figure 1).

### Case analysis

**Figure 2:**
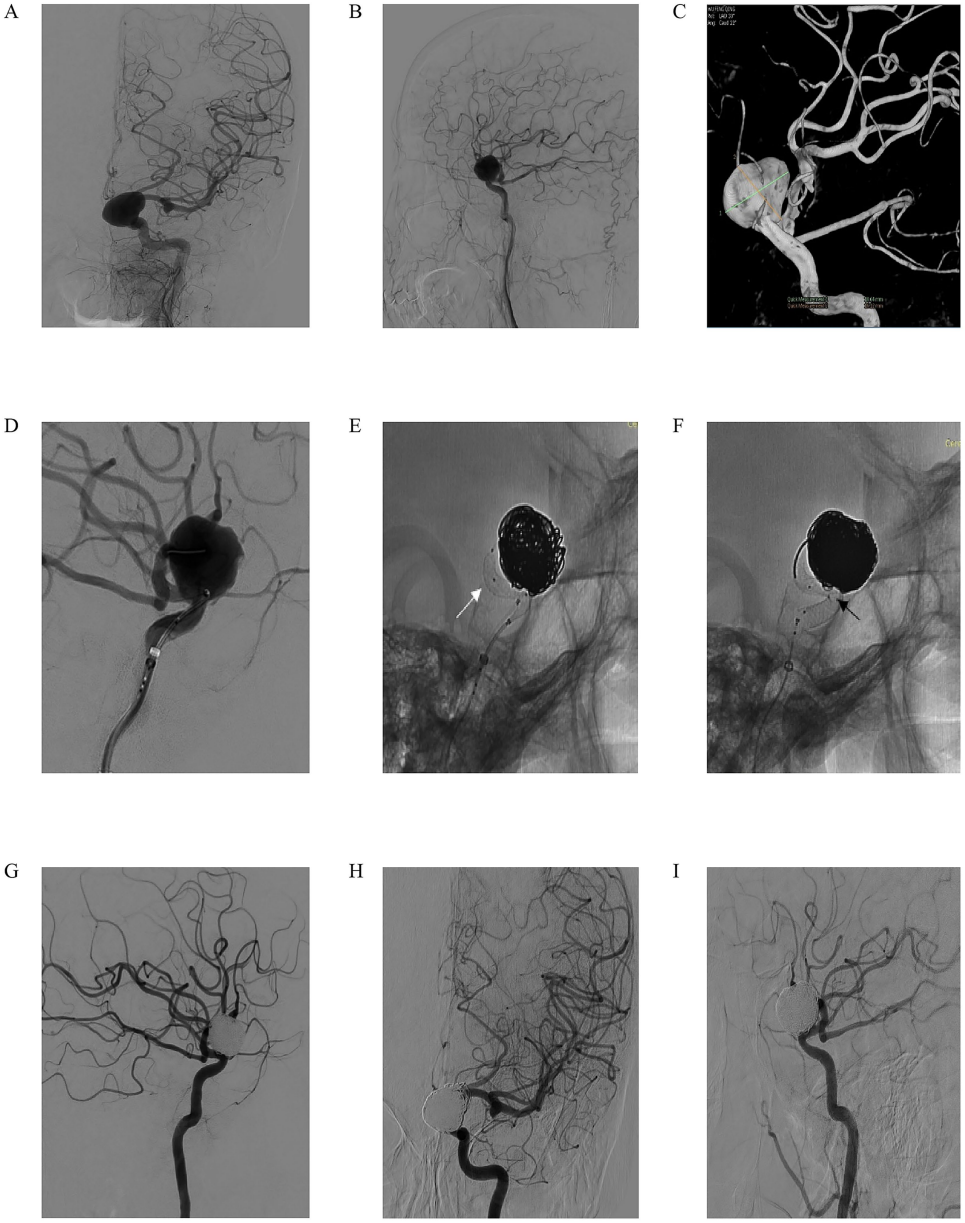
A 59-year-old woman reported a decrease in visual acuity of her left eye for 1 year, which had aggravated over the past 1 week. In the specialized physical examination, the movement of the eyeball was normal, the light reflex was normal, and the limb movement was normal. **(A,B)** Left ICA digital subtraction angiography (DSA) in anteroposterior **(A)** and lateral **(B)** views demonstrating a giant saccular aneurysm with a prominent “jet sign” (arrow), indicative of hemodynamic stress. **(C)** Three-dimensional DSA reconstruction confirming the irregular morphology of the giant aneurysm, measuring 18.64 mm × 16.22 mm; **(D)** Working-angle projection during intervention guiding the advancement of an intermediate catheter and a microcatheter (XT-27) to the middle cerebral artery; **(E)** The Lattice FD-MB deployed in a semi-released state (white arrow indicating the characteristic “lantern-shaped” configuration), allowing for partial coiling of the aneurysm sac; **(F)** Full deployment of the Lattice FD-MB, with black arrowheads confirming optimal wall apposition at critical vascular bends; **(G)** Immediate post-procedural DSA showing complete exclusion of the aneurysm from circulation with no evidence of contrast filling; **(H,I)** Four-month follow-up DSA in anteroposterior **(H)** and lateral **(I)** views confirming complete aneurysm occlusion, patency of the parent artery without stenosis, and successful hemodynamic reconstruction.

We present the case of a 59-year-old woman who presented with progressive left eye vision loss over 1 year, which worsened in the past week. Neurological examination showed normal ocular movements, intact pupillary light reflexes, and preserved limb mobility. Imaging evaluation included L-ICA DSA in anteroposterior and lateral views (Figures 2A,B), which revealed a saccular aneurysm in the C5-6 segment with a prominent “jet sign,” an indicator of hemodynamic stress. A 3D-DSA reconstruction (Figure 2C) confirmed an irregularly shaped giant aneurysm measuring 18.64 mm × 16.22 mm. During endovascular intervention, a working-angle projection (Figure 2D) guided the advancement of an intermediate catheter and the microcatheter XT27 to the middle cerebral artery. The Lattice FD-MB was deployed in a semi-released state (Figure 2E, white arrow indicating the “lantern-shaped” configuration within the Lattice), allowing partial coiling of the aneurysm sac. Following full deployment of the Lattice FD-MB (Figure 2F, black arrowheads confirming optimal wall apposition at vascular bends), immediate post-procedural DSA (Figure 2G) confirmed complete exclusion of the aneurysm from circulation, with no contrast filling. At the 4-month follow-up, DSA (Figures 2H,I) demonstrated complete aneurysm occlusion, patency preservation of the parent artery without stenosis, and successful hemodynamic reconstruction. This case illustrates the efficacy of Lattice FD-MB in achieving anatomical cure for complex giant aneurysms while preserving distal perfusion, with favorable short-term outcomes.

**Figure 3:**
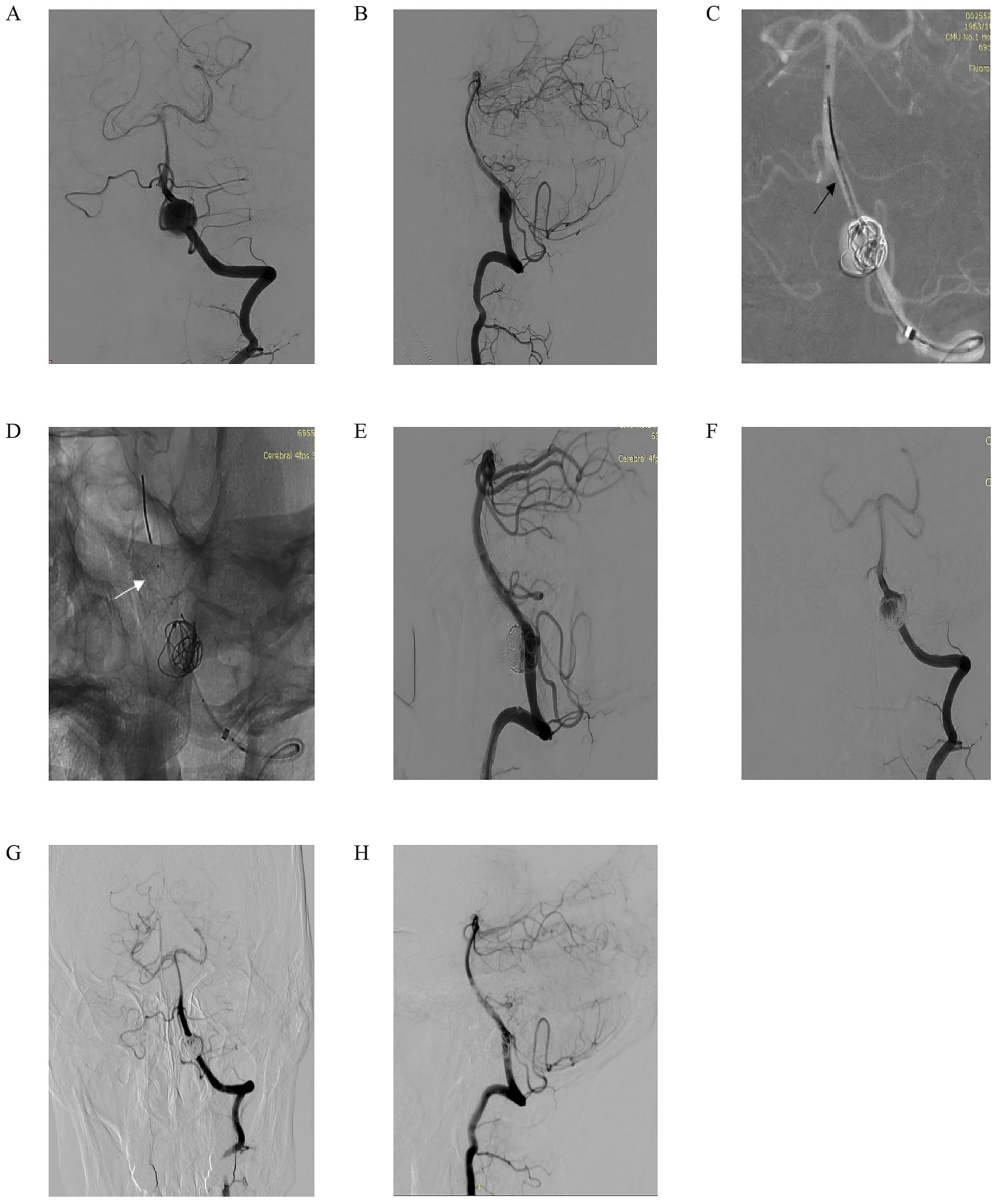
A 60-year-old man intermittently experienced headaches for 2 months. There was no nausea or vomiting, and no choking while drinking water. Computed tomography angiography (CTA) indicated an aneurysm in the left V4 segment. No positive signs were found in the specialized physical examination. **(A,B)** Left vertebral artery DSA in anteroposterior **(A)** and lateral **(B)** views confirming the presence of a fusiform aneurysm at the V4 segment; **(C)** Working-angle roadmap guidance for navigation of a 6F intermediate catheter and microcatheter (XT-27) to the target site. The black arrow marks the distal deployment landmark of the Lattice FD-MB; **(D)** Partial coiling of the aneurysm sac with the Lattice FD-MB maintained in a semi-deployed state. The white arrow highlights the optimal distal expansion of the device; **(E,F)** Post-coiling DSA in anteroposterior **(E)** and lateral **(F)** views demonstrating substantial aneurysm filling with coils, stable expansion of the Lattice FD-MB, preserved hemodynamics, and unimpaired flow into the left posterior inferior cerebellar artery (PICA); **(G,H)** Seven-month follow-up DSA in anteroposterior **(G)** and lateral **(H)** views confirming complete aneurysm occlusion, patency of the parent vessel without stenosis, and maintained perfusion of the PICA.

We present another case of a 60-year-old woman who presented with intermittent headaches for 2 months, without nausea, vomiting, or dysphagia. Computed tomography angiography (CTA) revealed a left vertebral artery V4 segment aneurysm, and neurological examination showed no focal deficits. Left vertebral artery DSA in anteroposterior and lateral views (Figures 3A,B) confirmed a fusiform aneurysm at the V4 segment. Under working-angle roadmap guidance (Figure 3C), a 6F intermediate catheter and microcatheter XT27 were navigated to the target site, with the Lattice FD-MB positioned distally (black arrow marks the deployment landmark). Partial coiling of the aneurysm sac was performed, while the Lattice FD-MB remained in a semi-deployed state (Figure 3D, white arrow highlights optimal distal expansion of the device). Post-coiling DSA (Figures 3E,F) demonstrated substantial aneurysm filling with coils, stable expansion of the Lattice FD-MB, preserved hemodynamics, and unimpaired flow in the left posterior inferior cerebellar artery (PICA). At the 7-month follow-up, DSA (Figures 3G,H) confirmed complete aneurysm occlusion and patency of the parent vessel without stenosis and maintained PICA perfusion. This case highlights the efficacy of combining flow diversion and coiling in managing complex fusiform V4 aneurysms, achieving durable occlusion while protecting critical branch vessels.

## Discussion

Recent advancements in non-invasive imaging and expanded screening programs have significantly improved the detection rates of UIA. Nevertheless, the treatment of anatomically complex aneurysms continues to pose substantial clinical challenges. This study demonstrates that the Lattice FD-MB achieves high procedural success, low complication rates, and progressive aneurysm occlusion in the treatment of UIA. The mechanical balloon-assisted design of the device enables precise deployment and optimal wall apposition, overcoming key challenges of traditional flow diverters in complex anatomy. This innovative design yielded several key outcomes as follows: (1) technical performance: 100% procedural success with no intraoperative device failures, establishing exceptional reliability; (2) safety profile: low intraoperative complication rate (6.9%, *n* = 2), all transient and clinically manageable, with 93.1% of patients (*n* = 27) maintaining optimal postoperative status without neurological deficits; and (3) functional outcomes: immediate post-procedural functional independence (mRS 0) in 79.3% of cases, with progressive improvement to 89.7% (mRS 0) at 12-month follow-up. The key advantage of the Lattice FD-MB lies in its integration of mechanical balloons for real-time controlled expansion, ensuring stable positioning even in tortuous or stenotic vessels. This is an improvement over conventional flow diverters prone to incomplete expansion.

Conventional endovascular approaches for UIAs, such as coil embolization, face significant challenges in managing complex aneurysms. The challenges include high recanalization rates, coil compaction, and parent artery compromise, particularly in wide-necked or tortuous lesions (15, 16). In contrast, the Lattice FD-MB integrates a mechanically expandable balloon system with a high-density nitinol mesh stent, enabling real-time controlled deployment, improved wall apposition, and precise parent vessel alignment. This design effectively mitigates the risks of incomplete scaffold expansion or device migration in anatomically challenging vascular segments, addressing key shortcomings of traditional flow diverters. To contextualize our findings, we compared key outcomes (procedural success, complication rates, and long-term functional recovery) with data from published studies on conventional UIA treatments (surgical clipping, endovascular coiling, and traditional flow diverters). The results are as follows: (1) procedural success rate: our 100% technical success rate aligns with reports on modern flow diverters but exceeds success rates in complex aneurysm cases treated with traditional coiling (17, 18), where wide necks or irregular morphologies often hinder complete deployment; (2) intraoperative complications: our 6.9% intraoperative complication rate (2/29) is lower than that reported for surgical clipping (19, 20) and comparable to endovascular coiling (21), where incomplete wall apposition increases procedural risks; (3) long-term functional outcomes: the 12-month functional independence rate of 89.7% (mRS 0) in our cohort outperforms surgical clipping (22), is comparable to results from high-volume coiling centers (22), and shows a steeper improvement trajectory; and (4) aneurysm occlusion durability: while our mid-term data (12 months) show promising trends, direct comparison with coiling’s 6.6–15.2% recanalization rate in complex UIAs (23) and traditional flow diverters’ 70%+ occlusion rate at 1 year (24) suggest that the Lattice FD-MB can balance durability and safety, particularly in anatomically challenging cases.

The indications for Lattice FD-MB include the following: (1) aneurysm type: unruptured saccular or fusiform aneurysms in adults; (2) anatomical sites: ICA (petrous segment to terminus) or vertebral artery; (3) morphological criteria: wide-necked aneurysms (neck width ≥4 mm or dome-to-neck ratio <2); and (4) parent vessel diameter: 2.0–5.6 mm. The contraindications include the following: (1) hypersensitivity to antiplatelet/anticoagulant agents or device materials; (2) active systemic or localized infection; and (3) contraindications to iodinated contrast agents. These parameters reflect broad applicability, with device diameters (2.3–5.0 mm) and lengths (13–50 mm) accommodating diverse anatomical configurations, while optimized radial force and flexibility enhance procedural success in tortuous cerebrovascular anatomy. This study observed a generally favorable prognosis for patients treated with the Lattice FD-MB for UIAs, though the outcomes varied (aneurysm shrinkage, no change in aneurysm size, in-stent stenosis, and aneurysm enlargement). These variations suggest that multiple factors influence prognosis, which can be analyzed from two perspectives: patient-related conditions and aneurysm characteristics.

Regarding patient-related factors, underlying conditions such as hypertension, dyslipidemia, and smoking history significantly impact the treatment process and outcomes. In hypertensive patients, prolonged high blood pressure imposes excessive stress on vascular walls, causing structural/functional changes (thickening, hardening, and loss of elasticity) that directly reduce the Lattice FD-MB apposition and stent stability (25, 26). Hypertension also promotes endothelial dysfunction and platelet aggregation, thereby increasing the risks of in-stent thrombosis and stenosis (27). Dyslipidemia, particularly elevated LDL-C, contributes to atherosclerotic plaque formation, narrowing of the lumens, impaired blood flow, and difficulties in stent deployment. Unstable plaques may rupture, triggering thrombus formation and compromising the outcomes (28). Smoking further damages endothelial cells, disrupting bioactive substance secretion, inducing vasoconstriction, enhancing platelet aggregation, and increasing oxidative stress, all of which increase the risk of in-stent stenosis (29–31). Notably, the patient with in-stent stenosis in this study had a smoking history, strongly linking smoking to impaired treatment efficacy via endothelial damage. From the perspective of aneurysm characteristics, morphology and location are crucial. Aneurysms in complex anatomical regions (e.g., vascular bifurcations or sharp angles) experience irregular hemodynamic forces, with rapid changes in blood flow direction/velocity, creating vortices and shear stress (32). This abnormal environment interferes with Lattice FD-MB deployment and apposition, limiting full coverage of the aneurysm neck (33–35) and suboptimal remodeling of intra-aneurysmal blood flow, thereby influencing outcomes.

Progressive increases were observed in the proportion of patients achieving mRS 0 (from discharge to follow-up) after Lattice FD-MB treatment for UIAs. Initial mRS 1–2 rates (20.7% at discharge) did not indicate treatment failure but reflected pre-existing mild neurological deficits (3 patients) and transient post-procedural effects (e.g., contrast-related symptoms, temporary nerve irritation). The reduction in these rates (to 10.3% at 12 months) aligns with the device’s ability to stabilize vascular anatomy and resolve acute perturbations, emphasizing that “disability” in these cases was mostly temporary and unrelated to major adverse events. The underlying mechanisms of this clinical efficacy involve four interrelated pathways described below: (1) precisely implanted in the parent artery, the Lattice FD-MB redistributes intra-aneurysmal blood flow. Its 3D lattice configuration diverts inflow jets, reducing turbulent shear stress in the aneurysm sac. This hemodynamic modulation promotes thrombus formation, followed by collagen deposition and fibroblast proliferation, leading to structural reinforcement and volumetric reduction (36); (2) the device’s optimized wall apposition design achieves dual functionality: blood flow diversion and preservation of physiological curvature via controlled radial force (37). Endothelialization begins by post-implantation day 7, with complete neointimal coverage by 3–6 months. This biological integration restores endothelial integrity and facilitates mechanotransduction-mediated alignment of vascular smooth muscle cells, restoring parent artery anatomy; (3) post-treatment hemodynamic stabilization improves the perianeurysmal microenvironment through three synergistic effects, which include restored cerebral perfusion that enhances mitochondrial bioenergetics in ischemic penumbra neurons, normalized shear stress that upregulates neuroangiogenic factors, and enhanced synaptic plasticity that drives functional reconfiguration of neural networks (38); and (4) the acute-phase inflammatory response, mediated by IL-10 and other anti-inflammatory cytokines, clears damage-associated molecular patterns. In the subacute phase, M2 microglial polarization dominates, suppressing complement activation and creating an anti-inflammatory milieu. By the chronic phase (>3 months), neuroinflammation resolves, eliminating secondary neuronal injury (39). This multidimensional framework explains the sustained improvement in mRS scores, highlighting the Lattice FD-MB’s unique capacity to synchronize biomechanical, biological, and neurological repair processes.

This study has several methodological limitations that warrant cautious interpretation. The limitations are as follows: (1) as an observational analysis from a single tertiary center, it introduces potential selection bias, particularly regarding the inclusion of anatomically favorable aneurysms; (2) the relatively short follow-up duration may not capture delayed complications or long-term outcomes. Intracranial aneurysm treatments, especially with flow diverters, can involve late events (e.g., aneurysm recanalization, progressive stent stenosis, and neurocognitive changes). Our 12-month data provide valuable midterm insights but do not fully assess occlusion durability or risks of delayed adverse events. Longer follow-up is needed to confirm sustained efficacy and safety, particularly for complex aneurysms prone to late recanalization; (3) the absence of a concurrent control group limits direct comparisons with alternative treatments, though we addressed this by contextualizing results against published data on surgical clipping, endovascular coiling, and traditional flow diverters, highlighting potential advantages of the Lattice FD-MB in complex UIAs; and (4) the retrospective design restricted multivariable analysis of critical prognostic determinants.

This comprehensive analysis supports the feasibility and safety of Lattice FD-MB in treating UIAs, with favorable patient prognoses. The use of the RROC and independent neuroradiologist review ensured a consistent and objective assessment of aneurysm occlusion, strengthening the reliability of observed trends toward progressive occlusion. While this study demonstrates the device’s feasibility, safety, and short-to-midterm efficacy, longer-term follow-up is necessary to evaluate occlusion durability and delayed complications, which will further inform its clinical utility.

## Data Availability

The original contributions presented in the study are included in the article/supplementary material, further inquiries can be directed to the corresponding author.
